# 2,4-Dimeth­oxy-6-[(*E*)-2-(4-meth­oxy­phenyl)ethen­yl]benzaldehyde

**DOI:** 10.1107/S1600536813007964

**Published:** 2013-04-05

**Authors:** Xiao-Lin Ge, Qiu-Xiang Guan, Sheng-Song Deng, Ban-Feng Ruan

**Affiliations:** a School of Medical Engineering, Hefei University of Technology, Hefei 230009, People’s Repulic of China

## Abstract

There are two conformationally similar mol­ecules in the asymmetric unit of he title compound, C_18_H_18_O_4_, in which the dihedral angles between the benzene rings are 23.54 (12) and 31.11 (12)°. In the crystal, C—H⋯π inter­actions (minimum H⋯ring centroid distance = 2.66 Å) link the mol­ecules into a layered structure extending down *a*.

## Related literature

The title compound is a derivative of the natural product resveratrol (*trans*-3,4,5-trihy­droxy­stilbene). For background to resveratrol, see: Jang *et al.* (1997 ▶); Orsini *et al.* (1997 ▶); Pettit *et al.* (2002 ▶). For standard bond lengths and angles, see: Boumendjel *et al.* (2008 ▶). For the synthesis of the title compound, see: Huang *et al.* (2007 ▶).
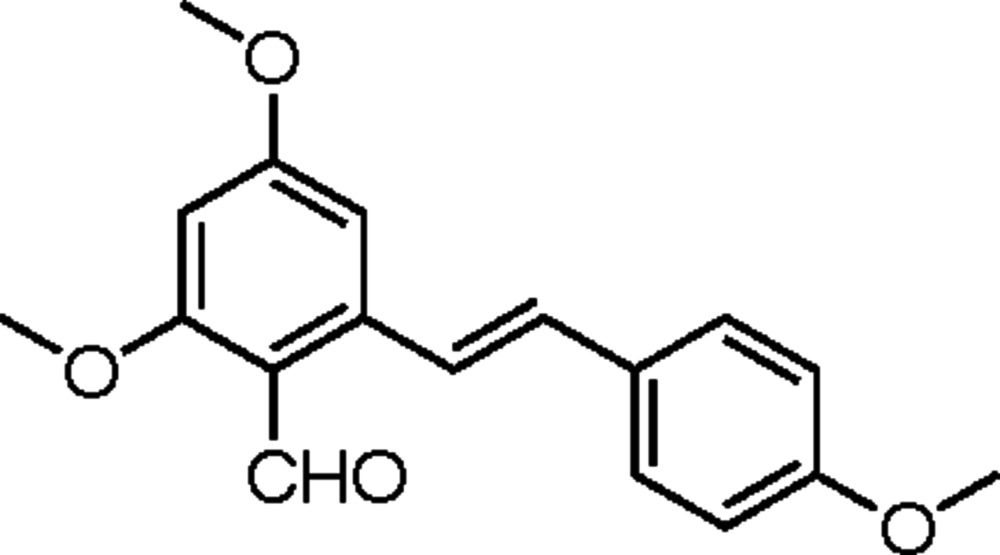


## Experimental

### 

#### Crystal data

C_18_H_18_O_4_
*M* *_r_* = 298.32
Triclinic, 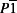

*a* = 9.292 (5) Å
*b* = 9.448 (5) Å
*c* = 17.547 (5) Å
α = 84.097 (5)°
β = 84.040 (5)°
γ = 83.315 (5)°
*V* = 1515.3 (12) Å^3^
*Z* = 4
Mo *K*α radiation
μ = 0.09 mm^−1^
*T* = 298 K
0.30 × 0.20 × 0.20 mm

#### Data collection

Bruker SMART CCD area-detector diffractometer
Absorption correction: multi-scan (*SADABS*; Bruker, 2000 ▶) *T*_min_ = 0.973, *T*_max_ = 0.982
10535 measured reflections
5272 independent reflections
3060 reflections with *I* > 2σ(*I*)
*R*_int_ = 0.039

#### Refinement

*R*[*F* ^2^ > 2σ(*F* ^2^)] = 0.048
*wR*(*F* ^2^) = 0.168
*S* = 0.79
5272 reflections
404 parameters
H-atom parameters constrained
Δρ_max_ = 0.15 e Å^−3^
Δρ_min_ = −0.18 e Å^−3^

### 

Data collection: *SMART* (Bruker, 2000 ▶); cell refinement: *SAINT* (Bruker, 2000 ▶); data reduction: *SAINT*; program(s) used to solve structure: *SHELXS97* (Sheldrick, 2008 ▶); program(s) used to refine structure: *SHELXL97* (Sheldrick, 2008 ▶); molecular graphics: *SHELXTL* (Sheldrick, 2008 ▶); software used to prepare material for publication: *SHELXTL*.

## Supplementary Material

Crystal structure: contains datablock(s) global, I. DOI: 10.1107/S1600536813007964/zs2252sup1.cif

Structure factors: contains datablock(s) I. DOI: 10.1107/S1600536813007964/zs2252Isup2.hkl

Supplementary material file. DOI: 10.1107/S1600536813007964/zs2252Isup3.ps

Supplementary material file. DOI: 10.1107/S1600536813007964/zs2252Isup4.mol

Supplementary material file. DOI: 10.1107/S1600536813007964/zs2252Isup5.cml

Additional supplementary materials:  crystallographic information; 3D view; checkCIF report

## Figures and Tables

**Table 1:** Hydrogen-bond geometry (Å, °) *Cg*2 and *Cg*4 are the centroids of the C12–C17 and C30–C35 rings, respectively.

*D*—H⋯*A*	*D*—H	H⋯*A*	*D*⋯*A*	*D*—H⋯*A*
C14—H14⋯*Cg*4^i^	0.93	2.88	3.597 (3)	135
C17—H14⋯*Cg*4^ii^	0.93	2.76	3.471 (3)	134
C32—H14⋯*Cg*2	0.93	2.82	3.530 (3)	135
C15—H14⋯*Cg*2^iii^	0.93	2.66	3.371 (3)	134

Symmetry codes: (i) 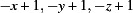; (ii) 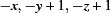; (iii) 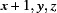.

**Fig. 1.**
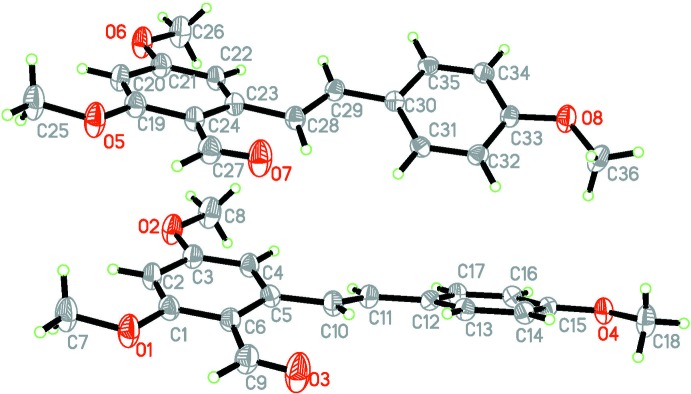
The molecular structure and atom numbering scheme for the two independent molecules of the title compound in the asymmetric unit, showing 30% probability displacement ellipsoids.

**Fig. 2.**
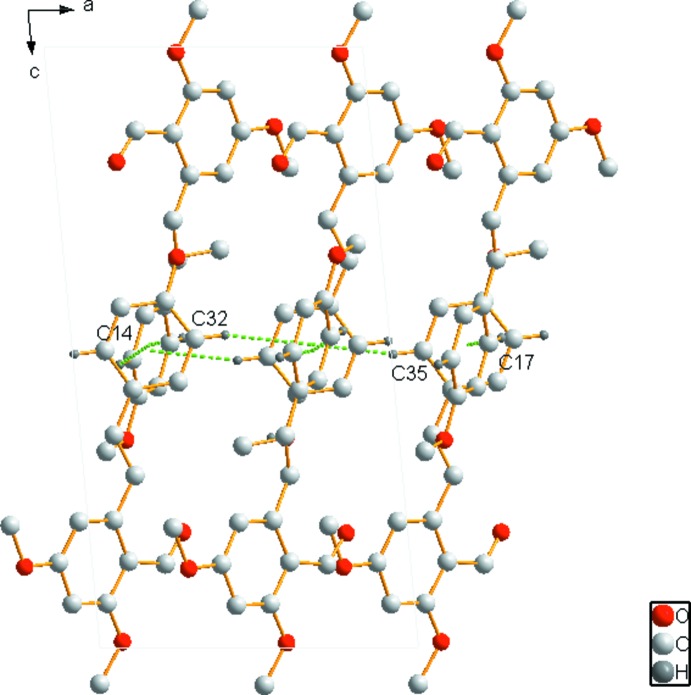
The layered structure of title compound formed through C—H···pi stacking interactions. Non-interactive H-atoms are omitted.

## supplementary crystallographic information

### Comment

Resveratrol (*trans*-3,4,,5-trihydroxystilbene), a naturally occurring phytoalexin present in medicinal plants, grape skin, peanuts and red wine, has attracted a great deal of attention because it has been suggested as a possible cancer chemopreventive agent on the basis of its inhibitory effects on tumor initiation, promotion, and progression (Jang *et al.*, 1997; Orsini *et al.*, 1997; Pettit *et al.*, 2002). In order to discover novel antitumor agents with high efficiency, a broad spectrum and safety, several series of derivatives of resveratrol were prepared in our laboratory. As an important intermediate, the title compound 2,4-dimethoxy-6-[(*E*)-2-(4-methoxyphenyl)ethenyl]benzaldehyde, was synthesized by the reaction of resveratrol using the Vilslmeier reaction and herein we report the crystal structure.

The title compound, C18 H18 O4, crystallizes in the triclinic space group *P*-1 with two independent molecules in the asymmetric unit (Fig. 1). All bond lengths are within normal ranges (Boumendjel *et al.*, 2008). The C10—C11 and C28—C29 bond lengths [1.319 (3) and 1.323 (3) Å] are consistent with the expected value for a C—C double bond and similarly, the C7—O1 and C25—O5 bond lengths [1.196 (3) and 1.210 (3) Å] are consistent with the expected value for a carbonyl C—O double bond. The dihedral angle between the two phenyl rings in each are 23.54 (12)° [C1–C6 (ring 1) and C12–C17) (ring 2)] and 31.11 (12)° [C19–C24 (ring 3) and C30–C35 (ring 4)]. As such, the structural differences between the two molecules in the title compound are only marginal.

In the crystal, the two molecules associate through C—H···pi stacking interactions [C14—H14···Cg(4)^i^; C17—H···Cg(4)^ii^; C32—H···Cg(2)^iii^; C35—H···Cg(2)^iv^: H···Cg = 2.88, 2.76, 2.82, 2.66 Å respectively] [for symmetry codes: (i) -*x* + 1, -*y* + 1, -*z* + 1; (ii) -*x* + 1, -*y*, -*z* + 1; (iii) *x*, *y*, *z*; (iv) *x* + 1, *y*, *z*]. Intermolecular methoxy C26—H···O8^v^ interactions [3.041 (4) Å] across an inversion centre generate a sheet structure (Fig. 2) [for symmetry code (v): -*x* + 2, -*y*, -*z* + 1]. Present also in the structure are intramolecular C—H···O interactions between the aldehyde and ethylenic donors and methoxy O-atom acceptors (Table 1).

### Experimental

Title compound was synthesized according to the reported method (Huang *et al.*, 2007). Crystals suitable for X-ray structure analysis were obtained by slow evaporation of a solution of the title compound in a petroleum ether/ dichloromethane mixture (1:1, *v*/*v*) at room temperature.

### Refinement

All the H atoms were placed in geometrically idealized positions and constrained to ride on their parent atoms, with C—H distances of 0.93–0.96 Å, and with *U*_iso_(H) = 1.2*U*_eq_(carrier) or 1.5*U*_eq_(methyl groups).

### Crystal data

**Table:**

C_18_H_18_O_4_	*Z* = 4
*M**_r_* = 298.32	*F*(000) = 632
Triclinic, *P*1	*D*_x_ = 1.308 Mg m^−^^3^
Hall symbol: -P 1	Mo *K*α radiation, λ = 0.71069 Å
*a* = 9.292 (5) Å	Cell parameters from 1909 reflections
*b* = 9.448 (5) Å	θ = 2.2–26.7°
*c* = 17.547 (5) Å	µ = 0.09 mm^−^^1^
α = 84.097 (5)°	*T* = 298 K
β = 84.040 (5)°	Block, colourless
γ = 83.315 (5)°	0.30 × 0.20 × 0.20 mm
*V* = 1515.3 (12) Å^3^	

### Data collection

**Table:**

Bruker SMART CCD area-detector diffractometer	5272 independent reflections
Radiation source: fine-focus sealed tube	3060 reflections with *I* > 2σ(*I*)
Graphite monochromator	*R*_int_ = 0.039
φ and ω scans	θ_max_ = 25.0°, θ_min_ = 2.2°
Absorption correction: multi-scan (*SADABS*; Bruker, 2000)	*h* = −11→10
*T*_min_ = 0.973, *T*_max_ = 0.982	*k* = −11→11
10535 measured reflections	*l* = −20→19

### Refinement

**Table:**

Refinement on *F*^2^	Secondary atom site location: difference Fourier map
Least-squares matrix: full	Hydrogen site location: inferred from neighbouring sites
*R*[*F*^2^ > 2σ(*F*^2^)] = 0.048	H-atom parameters constrained
*wR*(*F*^2^) = 0.168	*w* = 1/[σ^2^(*F*_o_^2^) + (0.1*P*)^2^ + 0.699*P*] where *P* = (*F*_o_^2^ + 2*F*_c_^2^)/3
*S* = 0.79	(Δ/σ)_max_ < 0.001
5272 reflections	Δρ_max_ = 0.15 e Å^−^^3^
404 parameters	Δρ_min_ = −0.18 e Å^−^^3^
0 restraints	Extinction correction: *SHELXL97* (Sheldrick, 2008), Fc^*^=kFc[1+0.001xFc^2^λ^3^/sin(2θ)]^-1/4^
Primary atom site location: structure-invariant direct methods	Extinction coefficient: 0.017 (2)

### Special details

**Table:**

Geometry. All e.s.d.'s (except the e.s.d. in the dihedral angle between two l.s. planes) are estimated using the full covariance matrix. The cell e.s.d.'s are taken into account individually in the estimation of e.s.d.'s in distances, angles and torsion angles; correlations between e.s.d.'s in cell parameters are only used when they are defined by crystal symmetry. An approximate (isotropic) treatment of cell e.s.d.'s is used for estimating e.s.d.'s involving l.s. planes.
Refinement. Refinement of *F*^2^ against ALL reflections. The weighted *R*-factor *wR* and goodness of fit *S* are based on *F*^2^, conventional *R*-factors *R* are based on *F*, with *F* set to zero for negative *F*^2^. The threshold expression of *F*^2^ > σ(*F*^2^) is used only for calculating *R*-factors(gt) *etc*. and is not relevant to the choice of reflections for refinement. *R*-factors based on *F*^2^ are statistically about twice as large as those based on *F*, and *R*- factors based on ALL data will be even larger.

### Fractional atomic coordinates and isotropic or equivalent isotropic displacement parameters (Å^2^)

**Table:**

	*x*	*y*	*z*	*U*_iso_*/*U*_eq_	
O4	0.14160 (19)	0.29091 (17)	0.65390 (9)	0.0490 (5)	
O8	0.6445 (2)	0.3074 (2)	0.65049 (10)	0.0584 (5)	
O2	0.6991 (2)	−0.0498 (2)	0.13171 (10)	0.0641 (6)	
O6	1.2124 (2)	−0.0612 (2)	0.13477 (10)	0.0663 (6)	
C12	0.3000 (2)	0.2119 (2)	0.42976 (13)	0.0377 (6)	
C15	0.1936 (2)	0.2736 (2)	0.57966 (13)	0.0368 (6)	
O5	0.9275 (2)	0.3222 (2)	0.00997 (10)	0.0698 (6)	
O1	0.4129 (2)	0.3330 (2)	0.00836 (10)	0.0674 (6)	
C33	0.6939 (3)	0.2778 (2)	0.57720 (13)	0.0393 (6)	
C29	0.8934 (3)	0.1922 (2)	0.35458 (13)	0.0413 (6)	
H29	0.9886	0.1486	0.3536	0.050*	
C13	0.2388 (2)	0.3479 (2)	0.44573 (13)	0.0400 (6)	
H13	0.2332	0.4200	0.4055	0.048*	
C11	0.3571 (3)	0.1739 (3)	0.35295 (14)	0.0444 (6)	
H11	0.4007	0.0807	0.3497	0.053*	
C30	0.8207 (3)	0.2192 (2)	0.43039 (13)	0.0367 (6)	
C35	0.8980 (3)	0.1892 (2)	0.49504 (13)	0.0404 (6)	
H35	0.9942	0.1490	0.4892	0.048*	
C31	0.6761 (3)	0.2748 (2)	0.44293 (13)	0.0394 (6)	
H31	0.6201	0.2922	0.4013	0.047*	
C16	0.2576 (3)	0.1382 (2)	0.56550 (13)	0.0401 (6)	
H16	0.2659	0.0670	0.6060	0.048*	
C4	0.5278 (3)	0.1016 (3)	0.20863 (14)	0.0463 (6)	
H4	0.5554	0.0505	0.2539	0.056*	
C19	0.9651 (3)	0.2384 (3)	0.07433 (14)	0.0491 (7)	
C24	0.8850 (3)	0.2751 (3)	0.14382 (14)	0.0436 (6)	
C3	0.5943 (3)	0.0623 (3)	0.13922 (15)	0.0471 (6)	
O7	0.7066 (2)	0.4560 (2)	0.19247 (11)	0.0734 (6)	
C32	0.6124 (3)	0.3051 (2)	0.51476 (13)	0.0410 (6)	
H32	0.5156	0.3435	0.5211	0.049*	
C34	0.8365 (3)	0.2172 (3)	0.56693 (14)	0.0443 (6)	
H34	0.8908	0.1954	0.6090	0.053*	
C23	0.9194 (3)	0.1940 (2)	0.21264 (13)	0.0421 (6)	
C20	1.0740 (3)	0.1273 (3)	0.07344 (15)	0.0522 (7)	
H20	1.1262	0.1047	0.0273	0.063*	
C1	0.4531 (3)	0.2517 (3)	0.07286 (14)	0.0473 (6)	
C5	0.4200 (3)	0.2160 (3)	0.21234 (13)	0.0430 (6)	
C2	0.5590 (3)	0.1378 (3)	0.07091 (14)	0.0516 (7)	
H2	0.6062	0.1118	0.0243	0.062*	
C28	0.8397 (3)	0.2227 (2)	0.28713 (14)	0.0444 (6)	
H28	0.7443	0.2654	0.2869	0.053*	
C21	1.1053 (3)	0.0493 (3)	0.14175 (15)	0.0481 (6)	
C14	0.1858 (3)	0.3803 (2)	0.51919 (13)	0.0395 (6)	
H14	0.1453	0.4726	0.5280	0.047*	
C6	0.3793 (3)	0.2931 (3)	0.14320 (14)	0.0441 (6)	
O3	0.1938 (3)	0.4673 (3)	0.19099 (12)	0.0913 (8)	
C17	0.3091 (3)	0.1078 (2)	0.49209 (13)	0.0396 (6)	
H17	0.3509	0.0157	0.4837	0.047*	
C22	1.0291 (3)	0.0813 (3)	0.21013 (14)	0.0475 (6)	
H22	1.0513	0.0267	0.2554	0.057*	
C10	0.3540 (3)	0.2567 (3)	0.28771 (14)	0.0492 (7)	
H10	0.3060	0.3484	0.2896	0.059*	
C27	0.7704 (3)	0.3951 (3)	0.13975 (16)	0.0545 (7)	
H27	0.7437	0.4282	0.0909	0.065*	
C18	0.0775 (3)	0.4283 (3)	0.67268 (16)	0.0655 (9)	
H18A	0.1483	0.4957	0.6614	0.098*	
H18B	0.0444	0.4241	0.7265	0.098*	
H18C	−0.0035	0.4581	0.6429	0.098*	
C9	0.2627 (3)	0.4111 (3)	0.13893 (17)	0.0593 (8)	
H9	0.2396	0.4467	0.0898	0.071*	
C25	1.0115 (4)	0.2970 (3)	−0.06044 (15)	0.0743 (9)	
H25A	1.1114	0.3084	−0.0557	0.111*	
H25B	0.9758	0.3642	−0.1008	0.111*	
H25C	1.0042	0.2014	−0.0725	0.111*	
C8	0.7351 (4)	−0.1367 (3)	0.19987 (16)	0.0722 (9)	
H8A	0.7697	−0.0791	0.2346	0.108*	
H8B	0.8097	−0.2118	0.1868	0.108*	
H8C	0.6502	−0.1779	0.2241	0.108*	
C36	0.5076 (3)	0.3858 (3)	0.66358 (16)	0.0632 (8)	
H36A	0.5030	0.4724	0.6297	0.095*	
H36B	0.4933	0.4091	0.7160	0.095*	
H36C	0.4329	0.3295	0.6540	0.095*	
C26	1.2461 (4)	−0.1482 (3)	0.20355 (16)	0.0727 (9)	
H26A	1.2762	−0.0899	0.2394	0.109*	
H26B	1.3232	−0.2215	0.1914	0.109*	
H26C	1.1613	−0.1917	0.2260	0.109*	
C7	0.4947 (4)	0.3057 (3)	−0.06241 (15)	0.0728 (9)	
H7A	0.5953	0.3159	−0.0586	0.109*	
H7B	0.4589	0.3728	−0.1028	0.109*	
H7C	0.4855	0.2101	−0.0738	0.109*	

### Atomic displacement parameters (Å^2^)

**Table:**

	*U*^11^	*U*^22^	*U*^33^	*U*^12^	*U*^13^	*U*^23^
O4	0.0641 (12)	0.0468 (10)	0.0339 (10)	0.0028 (9)	−0.0014 (9)	−0.0061 (8)
O8	0.0587 (12)	0.0752 (13)	0.0359 (11)	0.0159 (10)	−0.0024 (9)	−0.0079 (9)
O2	0.0753 (14)	0.0640 (12)	0.0428 (11)	0.0229 (11)	0.0027 (10)	0.0008 (9)
O6	0.0766 (14)	0.0687 (13)	0.0430 (11)	0.0281 (11)	0.0005 (10)	−0.0008 (9)
C12	0.0364 (13)	0.0377 (13)	0.0383 (14)	−0.0038 (10)	−0.0014 (11)	−0.0023 (10)
C15	0.0364 (13)	0.0404 (13)	0.0336 (14)	−0.0026 (11)	−0.0040 (11)	−0.0048 (10)
O5	0.0817 (14)	0.0805 (14)	0.0353 (11)	0.0236 (11)	0.0019 (10)	0.0076 (10)
O1	0.0782 (14)	0.0788 (14)	0.0348 (11)	0.0208 (11)	0.0007 (10)	0.0044 (9)
C33	0.0438 (15)	0.0380 (13)	0.0347 (14)	−0.0009 (11)	−0.0027 (11)	−0.0016 (10)
C29	0.0428 (14)	0.0406 (13)	0.0391 (15)	−0.0001 (11)	−0.0019 (12)	−0.0034 (11)
C13	0.0452 (15)	0.0379 (13)	0.0353 (14)	−0.0010 (11)	−0.0065 (11)	0.0039 (11)
C11	0.0488 (15)	0.0438 (14)	0.0392 (15)	−0.0006 (12)	−0.0005 (12)	−0.0062 (12)
C30	0.0395 (14)	0.0331 (12)	0.0371 (14)	−0.0032 (10)	−0.0033 (11)	−0.0022 (10)
C35	0.0334 (13)	0.0438 (14)	0.0440 (15)	−0.0008 (11)	−0.0076 (11)	−0.0035 (11)
C31	0.0423 (15)	0.0405 (13)	0.0350 (14)	−0.0011 (11)	−0.0105 (11)	0.0018 (10)
C16	0.0483 (15)	0.0328 (12)	0.0385 (14)	−0.0036 (11)	−0.0077 (12)	0.0028 (10)
C4	0.0518 (16)	0.0506 (15)	0.0336 (14)	0.0021 (13)	−0.0040 (12)	0.0007 (11)
C19	0.0574 (17)	0.0488 (15)	0.0383 (15)	0.0006 (13)	−0.0054 (13)	0.0030 (12)
C24	0.0491 (15)	0.0428 (14)	0.0368 (15)	0.0013 (12)	−0.0022 (12)	−0.0033 (11)
C3	0.0511 (16)	0.0482 (15)	0.0399 (15)	0.0024 (13)	−0.0018 (12)	−0.0050 (12)
O7	0.0889 (16)	0.0706 (13)	0.0507 (13)	0.0321 (12)	−0.0004 (11)	−0.0093 (10)
C32	0.0351 (14)	0.0435 (14)	0.0421 (15)	0.0026 (11)	−0.0037 (11)	−0.0012 (11)
C34	0.0406 (15)	0.0536 (15)	0.0386 (15)	0.0018 (12)	−0.0113 (12)	−0.0037 (12)
C23	0.0475 (15)	0.0406 (13)	0.0377 (14)	−0.0028 (12)	−0.0022 (12)	−0.0052 (11)
C20	0.0574 (17)	0.0561 (16)	0.0388 (16)	0.0063 (14)	0.0032 (13)	−0.0062 (13)
C1	0.0549 (16)	0.0515 (15)	0.0335 (14)	−0.0017 (13)	−0.0058 (12)	0.0026 (12)
C5	0.0472 (15)	0.0472 (14)	0.0351 (14)	−0.0050 (12)	−0.0052 (12)	−0.0047 (11)
C2	0.0611 (18)	0.0555 (16)	0.0344 (15)	0.0036 (14)	0.0016 (13)	−0.0035 (12)
C28	0.0486 (15)	0.0427 (14)	0.0403 (15)	0.0015 (12)	−0.0029 (12)	−0.0050 (11)
C21	0.0515 (16)	0.0460 (15)	0.0441 (16)	0.0047 (12)	−0.0025 (13)	−0.0051 (12)
C14	0.0439 (14)	0.0323 (12)	0.0412 (15)	0.0031 (11)	−0.0073 (11)	−0.0031 (11)
C6	0.0463 (15)	0.0476 (15)	0.0369 (15)	−0.0012 (12)	−0.0023 (12)	−0.0034 (11)
O3	0.1072 (18)	0.0979 (17)	0.0522 (14)	0.0503 (15)	0.0014 (13)	−0.0063 (12)
C17	0.0452 (14)	0.0311 (12)	0.0414 (15)	−0.0005 (10)	−0.0030 (11)	−0.0036 (10)
C22	0.0546 (16)	0.0511 (15)	0.0346 (15)	0.0019 (13)	−0.0033 (12)	−0.0035 (12)
C10	0.0545 (16)	0.0524 (15)	0.0390 (15)	0.0053 (13)	−0.0042 (12)	−0.0078 (12)
C27	0.0600 (18)	0.0560 (17)	0.0437 (16)	0.0056 (14)	−0.0046 (14)	−0.0004 (13)
C18	0.088 (2)	0.0557 (17)	0.0473 (18)	0.0156 (16)	0.0027 (16)	−0.0153 (14)
C9	0.0634 (19)	0.0636 (18)	0.0450 (17)	0.0100 (15)	0.0002 (15)	−0.0005 (14)
C25	0.091 (2)	0.086 (2)	0.0361 (17)	0.0159 (19)	0.0019 (16)	0.0065 (15)
C8	0.087 (2)	0.0664 (19)	0.0513 (19)	0.0222 (17)	−0.0011 (16)	0.0105 (15)
C36	0.0549 (18)	0.076 (2)	0.0524 (18)	0.0074 (15)	0.0112 (14)	−0.0083 (15)
C26	0.084 (2)	0.070 (2)	0.0526 (19)	0.0292 (17)	−0.0030 (16)	0.0004 (15)
C7	0.091 (2)	0.080 (2)	0.0374 (17)	0.0167 (18)	0.0009 (16)	0.0060 (15)

### Geometric parameters (Å, º)

**Table:**

O4—C15	1.361 (3)	C3—C2	1.380 (3)
O4—C18	1.420 (3)	O7—C27	1.206 (3)
O8—C33	1.365 (3)	C32—H32	0.9300
O8—C36	1.405 (3)	C34—H34	0.9300
O2—C3	1.360 (3)	C23—C22	1.386 (3)
O2—C8	1.426 (3)	C23—C28	1.468 (3)
O6—C21	1.362 (3)	C20—C21	1.380 (3)
O6—C26	1.430 (3)	C20—H20	0.9300
C12—C13	1.387 (3)	C1—C2	1.372 (3)
C12—C17	1.396 (3)	C1—C6	1.417 (3)
C12—C11	1.461 (3)	C5—C6	1.412 (3)
C15—C16	1.382 (3)	C5—C10	1.468 (3)
C15—C14	1.388 (3)	C2—H2	0.9300
O5—C19	1.363 (3)	C28—H28	0.9300
O5—C25	1.418 (3)	C21—C22	1.372 (3)
O1—C1	1.361 (3)	C14—H14	0.9300
O1—C7	1.418 (3)	C6—C9	1.463 (4)
C33—C32	1.382 (3)	O3—C9	1.194 (3)
C33—C34	1.383 (3)	C17—H17	0.9300
C29—C28	1.324 (3)	C22—H22	0.9300
C29—C30	1.461 (3)	C10—H10	0.9300
C29—H29	0.9300	C27—H27	0.9300
C13—C14	1.383 (3)	C18—H18A	0.9600
C13—H13	0.9300	C18—H18B	0.9600
C11—C10	1.319 (3)	C18—H18C	0.9600
C11—H11	0.9300	C9—H9	0.9300
C30—C31	1.389 (3)	C25—H25A	0.9600
C30—C35	1.392 (3)	C25—H25B	0.9600
C35—C34	1.368 (3)	C25—H25C	0.9600
C35—H35	0.9300	C8—H8A	0.9600
C31—C32	1.379 (3)	C8—H8B	0.9600
C31—H31	0.9300	C8—H8C	0.9600
C16—C17	1.374 (3)	C36—H36A	0.9600
C16—H16	0.9300	C36—H36B	0.9600
C4—C3	1.373 (3)	C36—H36C	0.9600
C4—C5	1.387 (3)	C26—H26A	0.9600
C4—H4	0.9300	C26—H26B	0.9600
C19—C20	1.371 (3)	C26—H26C	0.9600
C19—C24	1.414 (3)	C7—H7A	0.9600
C24—C23	1.407 (3)	C7—H7B	0.9600
C24—C27	1.464 (3)	C7—H7C	0.9600
			
C15—O4—C18	118.68 (19)	C1—C2—C3	119.0 (2)
C33—O8—C36	119.0 (2)	C1—C2—H2	120.5
C3—O2—C8	117.7 (2)	C3—C2—H2	120.5
C21—O6—C26	117.2 (2)	C29—C28—C23	124.8 (2)
C13—C12—C17	116.8 (2)	C29—C28—H28	117.6
C13—C12—C11	123.9 (2)	C23—C28—H28	117.6
C17—C12—C11	119.2 (2)	O6—C21—C22	124.1 (2)
O4—C15—C16	115.8 (2)	O6—C21—C20	114.9 (2)
O4—C15—C14	124.9 (2)	C22—C21—C20	121.0 (2)
C16—C15—C14	119.3 (2)	C13—C14—C15	119.3 (2)
C19—O5—C25	117.6 (2)	C13—C14—H14	120.4
C1—O1—C7	117.6 (2)	C15—C14—H14	120.4
O8—C33—C32	124.7 (2)	C5—C6—C1	118.2 (2)
O8—C33—C34	115.8 (2)	C5—C6—C9	124.4 (2)
C32—C33—C34	119.5 (2)	C1—C6—C9	117.4 (2)
C28—C29—C30	127.5 (2)	C16—C17—C12	121.5 (2)
C28—C29—H29	116.2	C16—C17—H17	119.2
C30—C29—H29	116.2	C12—C17—H17	119.2
C14—C13—C12	122.5 (2)	C21—C22—C23	120.9 (2)
C14—C13—H13	118.8	C21—C22—H22	119.6
C12—C13—H13	118.8	C23—C22—H22	119.6
C10—C11—C12	127.4 (2)	C11—C10—C5	125.5 (2)
C10—C11—H11	116.3	C11—C10—H10	117.3
C12—C11—H11	116.3	C5—C10—H10	117.3
C31—C30—C35	116.5 (2)	O7—C27—C24	127.4 (3)
C31—C30—C29	123.9 (2)	O7—C27—H27	116.3
C35—C30—C29	119.6 (2)	C24—C27—H27	116.3
C34—C35—C30	121.9 (2)	O4—C18—H18A	109.5
C34—C35—H35	119.0	O4—C18—H18B	109.5
C30—C35—H35	119.0	H18A—C18—H18B	109.5
C32—C31—C30	122.4 (2)	O4—C18—H18C	109.5
C32—C31—H31	118.8	H18A—C18—H18C	109.5
C30—C31—H31	118.8	H18B—C18—H18C	109.5
C17—C16—C15	120.6 (2)	O3—C9—C6	127.8 (3)
C17—C16—H16	119.7	O3—C9—H9	116.1
C15—C16—H16	119.7	C6—C9—H9	116.1
C3—C4—C5	121.1 (2)	O5—C25—H25A	109.5
C3—C4—H4	119.4	O5—C25—H25B	109.5
C5—C4—H4	119.4	H25A—C25—H25B	109.5
O5—C19—C20	123.4 (2)	O5—C25—H25C	109.5
O5—C19—C24	115.3 (2)	H25A—C25—H25C	109.5
C20—C19—C24	121.3 (2)	H25B—C25—H25C	109.5
C23—C24—C19	118.3 (2)	O2—C8—H8A	109.5
C23—C24—C27	123.8 (2)	O2—C8—H8B	109.5
C19—C24—C27	117.9 (2)	H8A—C8—H8B	109.5
O2—C3—C4	123.9 (2)	O2—C8—H8C	109.5
O2—C3—C2	114.9 (2)	H8A—C8—H8C	109.5
C4—C3—C2	121.1 (2)	H8B—C8—H8C	109.5
C31—C32—C33	119.3 (2)	O8—C36—H36A	109.5
C31—C32—H32	120.3	O8—C36—H36B	109.5
C33—C32—H32	120.3	H36A—C36—H36B	109.5
C35—C34—C33	120.2 (2)	O8—C36—H36C	109.5
C35—C34—H34	119.9	H36A—C36—H36C	109.5
C33—C34—H34	119.9	H36B—C36—H36C	109.5
C22—C23—C24	119.3 (2)	O6—C26—H26A	109.5
C22—C23—C28	118.8 (2)	O6—C26—H26B	109.5
C24—C23—C28	121.9 (2)	H26A—C26—H26B	109.5
C19—C20—C21	119.2 (2)	O6—C26—H26C	109.5
C19—C20—H20	120.4	H26A—C26—H26C	109.5
C21—C20—H20	120.4	H26B—C26—H26C	109.5
O1—C1—C2	122.8 (2)	O1—C7—H7A	109.5
O1—C1—C6	115.6 (2)	O1—C7—H7B	109.5
C2—C1—C6	121.5 (2)	H7A—C7—H7B	109.5
C4—C5—C6	119.0 (2)	O1—C7—H7C	109.5
C4—C5—C10	119.5 (2)	H7A—C7—H7C	109.5
C6—C5—C10	121.6 (2)	H7B—C7—H7C	109.5
			
C18—O4—C15—C16	178.6 (2)	C7—O1—C1—C6	173.1 (2)
C18—O4—C15—C14	−1.7 (3)	C3—C4—C5—C6	−0.2 (4)
C36—O8—C33—C32	−7.7 (4)	C3—C4—C5—C10	−178.5 (2)
C36—O8—C33—C34	171.8 (2)	O1—C1—C2—C3	179.1 (2)
C17—C12—C13—C14	−1.4 (3)	C6—C1—C2—C3	−0.2 (4)
C11—C12—C13—C14	−179.9 (2)	O2—C3—C2—C1	179.2 (2)
C13—C12—C11—C10	−3.6 (4)	C4—C3—C2—C1	−1.5 (4)
C17—C12—C11—C10	177.9 (2)	C30—C29—C28—C23	179.3 (2)
C28—C29—C30—C31	4.4 (4)	C22—C23—C28—C29	27.9 (4)
C28—C29—C30—C35	−175.4 (2)	C24—C23—C28—C29	−153.8 (2)
C31—C30—C35—C34	−2.1 (3)	C26—O6—C21—C22	0.1 (4)
C29—C30—C35—C34	177.7 (2)	C26—O6—C21—C20	−178.1 (3)
C35—C30—C31—C32	2.7 (3)	C19—C20—C21—O6	178.3 (2)
C29—C30—C31—C32	−177.0 (2)	C19—C20—C21—C22	0.1 (4)
O4—C15—C16—C17	177.7 (2)	C12—C13—C14—C15	0.0 (4)
C14—C15—C16—C17	−2.0 (3)	O4—C15—C14—C13	−178.0 (2)
C25—O5—C19—C20	−3.6 (4)	C16—C15—C14—C13	1.7 (3)
C25—O5—C19—C24	175.3 (2)	C4—C5—C6—C1	−1.4 (4)
O5—C19—C24—C23	−179.3 (2)	C10—C5—C6—C1	176.9 (2)
C20—C19—C24—C23	−0.3 (4)	C4—C5—C6—C9	176.9 (2)
O5—C19—C24—C27	1.1 (4)	C10—C5—C6—C9	−4.8 (4)
C20—C19—C24—C27	−179.9 (2)	O1—C1—C6—C5	−177.7 (2)
C8—O2—C3—C4	4.0 (4)	C2—C1—C6—C5	1.6 (4)
C8—O2—C3—C2	−176.8 (3)	O1—C1—C6—C9	3.8 (4)
C5—C4—C3—O2	−179.1 (2)	C2—C1—C6—C9	−176.8 (2)
C5—C4—C3—C2	1.7 (4)	C15—C16—C17—C12	0.6 (4)
C30—C31—C32—C33	−0.9 (3)	C13—C12—C17—C16	1.0 (3)
O8—C33—C32—C31	177.8 (2)	C11—C12—C17—C16	179.7 (2)
C34—C33—C32—C31	−1.7 (3)	O6—C21—C22—C23	−178.8 (2)
C30—C35—C34—C33	−0.4 (4)	C20—C21—C22—C23	−0.7 (4)
O8—C33—C34—C35	−177.2 (2)	C24—C23—C22—C21	0.8 (4)
C32—C33—C34—C35	2.3 (4)	C28—C23—C22—C21	179.1 (2)
C19—C24—C23—C22	−0.3 (4)	C12—C11—C10—C5	176.5 (2)
C27—C24—C23—C22	179.3 (2)	C4—C5—C10—C11	−19.5 (4)
C19—C24—C23—C28	−178.6 (2)	C6—C5—C10—C11	162.3 (3)
C27—C24—C23—C28	1.0 (4)	C23—C24—C27—O7	12.8 (5)
O5—C19—C20—C21	179.3 (3)	C19—C24—C27—O7	−167.6 (3)
C24—C19—C20—C21	0.4 (4)	C5—C6—C9—O3	6.5 (5)
C7—O1—C1—C2	−6.2 (4)	C1—C6—C9—O3	−175.2 (3)

### Hydrogen-bond geometry (Å, º)

Cg2 and Cg4 are the centroids of the C12–C17 and C30–C35 rings, respectively.

**Table:**

*D*—H···*A*	*D*—H	H···*A*	*D*···*A*	*D*—H···*A*
C9—H9···O1	0.93	2.29	2.681 (4)	105
C10—H10···O3	0.93	2.25	2.863 (4)	123
C27—H27···O5	0.93	2.31	2.681 (4)	103
C28—H28···O7	0.93	2.34	2.861 (3)	115
C14—H14···*Cg*4^i^	0.93	2.88	3.597 (3)	135
C17—H14···*Cg*4^ii^	0.93	2.76	3.471 (3)	134
C32—H14···*Cg*2	0.93	2.82	3.530 (3)	135
C15—H14···*Cg*2^iii^	0.93	2.66	3.371 (3)	134

Symmetry codes: (i) −*x*+1, −*y*+1, −*z*+1; (ii) −*x*, −*y*+1, −*z*+1; (iii) *x*+1, *y*, *z*.
